# Indole Acetic Acid Exerts Anti-Depressive Effects on an Animal Model of Chronic Mild Stress

**DOI:** 10.3390/nu14235019

**Published:** 2022-11-25

**Authors:** Ying Chen, Peijun Tian, Zheng Wang, Ruili Pan, Kexin Shang, Gang Wang, Jianxin Zhao, Wei Chen

**Affiliations:** 1 State Key Laboratory of Food Science and Technology, Jiangnan University, Wuxi 214122, China; 2 School of Food Science and Technology, Jiangnan University, Wuxi 214122, China; 3 National Engineering Research Center for Functional Food, Jiangnan University, Wuxi 214122, China; 4 (Yangzhou) Institute of Food Biotechnology, Jiangnan University, Yangzhou 225004, China

**Keywords:** indole acetic acid, depression, HPA axis, serotonin, gut microbiota, indole derivative

## Abstract

Indole acetic acid (IAA), an intestinal bacteria-derived tryptophan metabolite, has been detected at abnormal concentrations in the cerebrospinal fluid and urine of depressed individuals. The effects of such altered IAA concentrations on mood regulation are not known. A mouse model of unpredictable chronic mild stress (UCMS) was used to assess the effects of IAA administration (50 mg/kg). Treatment with IAA for 5 weeks attenuated depression and anxiety-like behaviours, improved hypothalamus–pituitary–adrenal axis dysfunction and increased brain-derived neurotrophic factor expression. IAA supplementation also enhanced the serotonin pathway in the brain and gut. UCMS caused an imbalance of microbial indole metabolites in the colon, whereas IAA treatment reversed this. However, IAA intake did not affect the concentrations of indoles in the brain. Intestinal bacteria in different sections of the gut were altered by IAA treatment, with the colon showing more changes than other segments. The gut microbiome in the colon had increased proportions of *Ruminococcaceae* UCG013, *Ruminiclostridium* 6, *Prevotella*, *Alloprevotella* and *Bacteroides* species, which can produce short-chain fatty acids and indole derivatives. Cumulatively, our study highlights the potential of IAA treatment to alleviate mood disorders and offers a theoretical basis for understanding the antidepressant effects of IAA.

## 1. Introduction

Depression is a complex and debilitating psychiatric disorder that often manifests as continuous and prolonged sadness. The pathogenesis of depression involves biological, genetic, psychological and social factors [1]. The morbidity rate of depression is approximately 10% worldwide [2]. It can cause brain dysfunction such as cognitive impairment, memory deficit and anhedonia. Recent studies have implicated abnormalities in multiple systems in depressed individuals, namely, the serotonergic, noradrenergic, dopaminergic, cholinergic, glutamatergic and GABAergic systems; the corticotropic-releasing factor (CRF) and hypothalamus–pituitary–adrenal (HPA) axis; and neurophysiology [3]. Current antidepressant drugs, such as monoamine oxidase inhibitors, selective serotonin reuptake inhibitors, tricyclic agents and serotonin–norepinephrine reuptake inhibitors, act by increasing neurotransmitter concentrations but have notable side effects [4]. The development of new antidepressant therapeutic strategies has therefore attracted increasing scientific interest around the world.

Accumulating evidence has shown that the intestinal microbiota plays a vital role in ameliorating depression [5,6] and can influence critical physiological functions pertaining to immune system homeostasis, bacteria–host metabolism and even brain activity [7]. The composition and structure of intestinal bacteria vary in patients with major depressive disorder (MDD) and mouse models for depression; the phyla Bacteroidetes, Firmicutes and Verrucomicrobia and the genera *Lachnospiraceae*, *Turicibacter*, *Lactobacillus*, *Bifidobacterium*, *Streptococcus*, *Alistipes*, *Bacteroides*, *Prevotella* and *Akkermansia* are known to be altered in these cases [8]. Depressive behaviours and metabolite disruption in mice exposed to unpredictable chronic mild stress (UCMS) have been shown to be attenuated by supplementation with faecal microbiota from healthy donors [9]. Psychobiotics, largely isolated from bacteria such as *Lactobacillus* and *Bifidobacterium* strains in healthy hosts, have been reported to alleviate depression and anxiety-like behaviours in mice and depressive symptoms in patients with MDD by altering the composition of the gut microbiome [10,11].

Tryptophan, an essential amino acid, is a biosynthetic precursor of many microbial and host metabolites known to mediate diseases. Specially, the serotonin pathway of tryptophan metabolism is a key factor that has been shown to modulate depression. Microbial tryptophan metabolites contain indole and indole derivatives. An imbalance in tryptophan metabolism caused by intestinal microorganisms has been observed in depressed patients and animals [12,13]. Dietary tryptophan supplementation alleviated emotional behaviours and regulated inflammatory responses in chronically stressed mice, indicating an association between indole derivatives and depressive behaviours [14]. Indole administration also induced overactivation of the adrenal medulla and increased the emotional responses in UCMS-exposed mice [15,16]. However, another study demonstrated that indole alters neurogenesis, which has been shown to be negatively correlated with depression [17,18]. Indole-3-propionic acid has also been shown to promote nerve regeneration and repair [19]. Indole acetic acid (IAA), a microbial tryptophan metabolite, is known to improve gut permeability and host immunity. Increasing evidence has indicated that indole metabolites affect mood regulation and neuroregulation. Altered concentrations of IAA have been detected in the urine and cerebrospinal fluid of patients with depression by metabolomics [20,21,22]. Despite this evidence, the effects of microbial IAA on chronic stress-induced depressive behaviours remain unknown.

Here, a mouse model of UCMS was used to explore the effects of IAA administration on depression- and anxiety-like, cognitive and social behaviours. Alterations of neurobiological factors and the gut microbiota resulting from IAA supplementation were also investigated.

## 2. Materials and Methods

### 2.1. Animal Design

Twenty-two male C57BL/6J mice (5 weeks old, 18–20 g of weight) were purchased from Weitonglihua Experimental Animal Tech Co. (Beijing, China). All of the animals were housed at 22 ± 3 °C under a 12 h light/dark cycle at the Jiangnan University Laboratory Animal Centre. The experiments were carried out in compliance with the Institutional Animal Care and Use Committee guidelines (Jiangsu, China). All of the protocols were approved by the Ethics Committee of Experimental Animals at Jiangnan University (JN. No20210530c1040730[133]).

**Figure 1:**
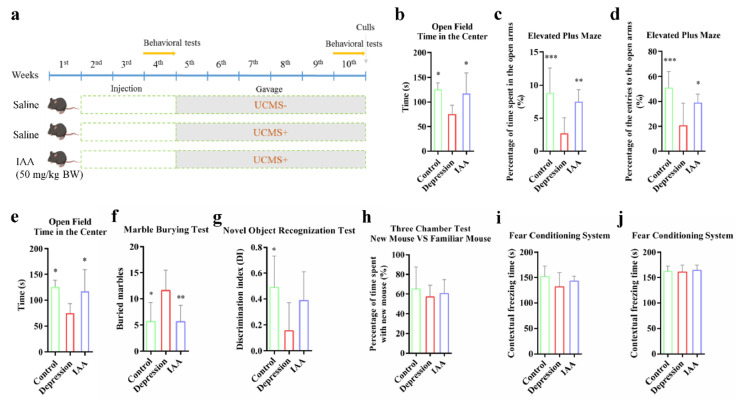
Experimental design and results of the behavioural tests. (**a**) Schedule of C57BL/6J mice treatment during 10 weeks. (**b**–**j**) Evaluation of anxiety-like, depression-like, social behaviours and cognition in mice treated with UCMS and IAA. One-way ANOVA with Tukey’s post-test was used. Compared with the depression group, * *p* < 0.05, ** *p* < 0.01, *** *p* < 0.001.

After acclimatisation for 1 week, the mice were randomly assigned to three groups in a completely randomized design (n = 6–8). The experimental schedule is described in Figure 1. To study the effects of IAA on the emotional behaviours of and cognition in healthy mice, a short-term test was performed. During the first 2 weeks, none of the groups were subjected to UCMS. These mice were intraperitoneally injected with either IAA (50 mg/kg body weight) (Sigma-Aldrich) or saline daily. In the fourth week, behavioural tests were conducted. Then, all of the mice, barring the control group, were subjected to various mild stressors for 5 weeks. The UCMS protocol was followed as previously described [23]; it comprised water or food deprivation, removal of bedding, wet bedding, crowding, continuous light, social isolation, restraint in a drilled tube, forced swimming and tail clipping (see Supplementary Materials). The stressors of the UCMS procedure were applied in a semi-random manner (Table S1), the sequence was changed every week. The IAA group mice were intragastrically administered IAA (50 mg/kg) to explore the role of intestinal IAA in mental disorders. A gavage of saline in the same volume as that of IAA was given to the other two groups. The behavioural experiments were conducted again after an intermission of 5 weeks.

### 2.2. Behavioural Assays

The details of the behavioural test are described in the Supplementary Materials. The mice were allowed to adapt to the testing room for 30 min before conducting the tests. After each round of tests, the equipment and other objects used were cleaned with 10% ethanol to avoid olfactory cuing. The behavioural tests conducted in a blinded fashion included marble burying (MB), novel object recognition (NOR), the three-chambered social approach task (TCS), the elevated plus maze (EPM), the open field test (OFT), the fear conditioning paradigm (FC) and forced swim (FS). Further parameter analysis of experimental videos was carried out using Ethovision software (version 3.1, Tracksys Ltd., Nottingham, UK).

### 2.3. UPLC-MS Analysis of Trp-Indole Derivatives and Neurotransmitters

The contents of the colon, plasma and hippocampus were collected. The sample preparation method has been described previously [24]. The samples were analysed using a DIONEX UltiMate 3000 HPLC apparatus equipped with a Q-Exactive mass spectrometer (Thermo Fisher Scientific, Waltham, MA, USA) and an ACQUITY UPLC^®^ BEH C18 column (1.7 µm, 100 mm × 2.10 mm, Waters, Milford, MA, USA). The sample volume injected was 2 µL. The solvent system consisted of 0.1% (*v*/*v*) formic acid (mobile phage A) and acetonitrile (mobile phage B) delivered at a flow rate of 0.3 mL/min. The gradient was as follows: 5% B, 0–3 min; 30% B, 3–9 min; 100% B, 9–15 min; 100% B, 15–16.5 min; and 5% B, 16.5–20 min. The mass spectrometer was operated in positive electrospray ionisation (ESI+) mode at a spray voltage of 3500 V. The heated capillary temperature was set to 320 °C. Nitrogen was used as the sheath and auxiliary gas at flow rates of 35 and 15 units, respectively.

The external standard method was used for quantification. The calibration curve of each analyte was plotted from 1 ppb to 10 ppm. All standards were obtained from Sigma-Aldrich.

### 2.4. SCFAs Analysis

The analysis of short-chain fatty acids (SCFAs) in the caecum was conducted using a modified version of a previously published method [25]. Briefly, approximately 40 mg of a freeze-dried sample was added to 0.5 mL of saturated NaCl solution. The mixture was violently homogenised (65 Hz) for 90 s. It was then supplemented with 40 µL of 10% H_2_SO_4_ and shaken. The acidified sample was mixed with 1 mL of anhydrous ether and centrifuged at 15,000× *g* for 15 min at 4 °C. The supernatant was then analysed by gas chromatography–mass spectrometry (GC–MS).

### 2.5. Quantitative Real-Time PCR

The extraction of total RNA from tissues was carried out using TRIzol (Invitrogen, Carlsbad, CA, USA). cDNA was obtained using a HiScript III 1st Strand cDNA synthesis kit (Vazyme, Nanjing, China). Transcripts were detected using SYBR Green Supermix and Bio-Rad CFX384 (Bio-Rad, Hercules, CA, USA). The fold changes in the mRNA transcripts of different genes were calculated by normalising to *Gapdh*. The primer sequences used are shown in Table S2.

### 2.6. Gut Microbiota Profiling

Metagenomic DNA was extracted from the colon and small intestine of the mice using a Fast Stool kit (MP Biomedicals LLC, Irvine, CA, USA). The 16S rRNA V3V4 region was amplified using paired primers (341F: 5′-CCTAYGGGRBGCASCAG-3′ and 806R: 5′-GGACTACNNGGGTATCTAAT-3′). Next, 500-bp DNA products were purified using a TIANgel Mini Purification kit (TIANGEN, Beijing, China). The purified samples were quantified and mixed using a Qubit dsDNA Assay kit (Life Technologies, Carlsbad, CA, USA). The mixed libraries were sequenced on the Illumina Miseq PE300 platform (Illumina, San Diego, CA, USA). The 16S rRNA sequences of the microbial community were then analysed using QIIME2. The amplicon sequence variants were rarefied to 5000 on the basis of sampling depth, and the diversity of gut bacteria from different intestinal segments was analysed.

### 2.7. Enzyme-Linked Immunosorbent Assay

The quantification of corticosterone (CORT) and adrenocorticotropic hormone (ACTH) in haemolysis-free blood plasma was conducted using an ELISA kit (SenBeiJia Biological Technology Co., Ltd., Nanjing, China). Total protein extracted from the hippocampus and hypothalamus samples were prepared in accordance with the instructions in the ELISA kits. Protein concentrations were determined using a Micro BCA Protein Assay kit (Sangon Biotech, Shanghai, China). The concentrations of c-Fos and brain-derived neurotrophic factor (BDNF) in the hippocampus and of CRF in the hypothalamus were measured and normalized with respect to total protein.

### 2.8. Statistical Analysis

The sample size was determined by preliminary experiments evaluating variance and effects. All values are presented as the mean ± standard deviation (SD). Data analysis was conducted using GraphPad Prism 6 (GraphPad Software, San Diego, CA, USA). The microbial data were analysed using QIIME2 and STAMP software. For multiple comparisons, one-way analysis of variance with Tukey’s *post hoc* means testing was conducted. Statistical significance is indicated as an adjusted *p* value < 0.05 compared with the UCMS model group. In the gut microbiota analysis, the false discovery rate (FDR) method was used to adjust the *p* value.

## 3. Results

### 3.1. IAA Alters the Anxiety- and Depression-Like Behaviours Induced by UCMS

Mice administered IAA via intraperitoneal injection for 2 weeks displayed no obvious differences from mice in other groups in tests of anxiety-like, depression-like and social behaviours and cognitive memory (Figure S1). This demonstrated that the dosage of IAA we used had no negative effects on emotional regulation. The mice were then exposed to UCMS for 5 weeks. Compared with the control group, UCMS exposure prominently decreased the time spent in the centre in the OFT. These mice also spent less time in the open arms of the EPM and demonstrated longer periods of immobility in the FS test than the control mice (Figure 1b–e). Supplementation with IAA prominently alleviated the depression and anxiety-like behaviours caused by UCMS. The IAA-treated mice also presented less stereotypes than the depression group (Figure 1f). To test memory, the NOR and FC tests were carried out. A dramatic decline in the discrimination index was observed in UCMS-treated mice, indicative of impaired ability to identify novel objects (Figure 1g). It appeared that the IAA treatment slowed the decline of cognitive memory. However, no differences were observed in memory of cues or context between the three groups (Figure 1i–j). Additionally, interactions with a familiar mouse and a new mouse were not significantly affected by UCMS exposure and IAA treatment (Figure 1h). These results indicate that IAA supplementation could effectively ameliorate the anxious and depressive behaviours induced by UCMS.

### 3.2. IAA Treatment Affects the HPA Axis and c-Fos/BDNF Expression

**Figure 2:**
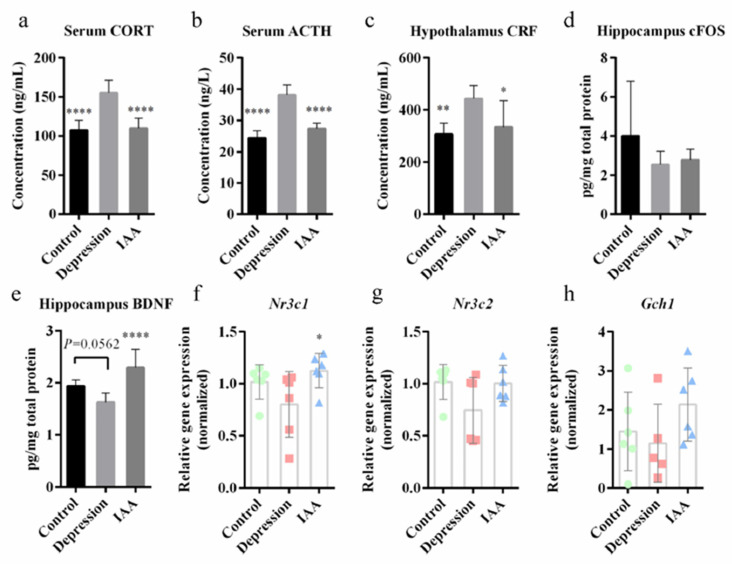
The effects of IAA on the HPA axis and c-Fos/BDNF expression. (**a**–**c**) Protein levels of CORT and ACTH in the serum and of CRF in the hypothalamus to evaluate the HPA axis response. (**d**–**e**) Expression of c-Fos and BDNF in the hippocampus. (**f**–**h**) Shown are the messenger RNA levels of *Nr3c1*, *Nr3c2* and *Gch1* gene in the hippocampus. Data are means and SD, compared with the depression group, * *p* < 0.05, ** *p* < 0.01, **** *p* < 0.0001, one-way ANOVA followed by Tukey’s post hoc test.

To explore the regulation of physiological responses to stress by the HPA axis, the production and secretion of CRF, ACTH and CORT were quantified. Compared with vehicle-treated controls, UCMS abnormally elevated the concentrations of these proteins (Figure 2a–c). Treating UCMS-exposed mice with IAA for 5 weeks decreased HPA axis overactivation relative to that in depressed mice. The IAA intervention notably increased the transcript levels of the glucocorticoid receptor (NR3C1) but not of the mineralocorticoid receptor (NR3C2) in the hippocampus (Figure 2f–g). Stress is known to be accompanied by abnormal c-Fos expression. A slight decrease in c-Fos was observed in the UCMS-treated mice, which was not alleviated by IAA treatment (Figure 2d). However, the IAA treatment significantly increased BDNF expression in the hippocampus (Figure 2e). These results suggest that IAA may alleviate depression by regulating the HPA axis and BDNF concentration.

### 3.3. Administration of IAA Regulates Dopamine and the Serotonergic System

**Figure 3:**
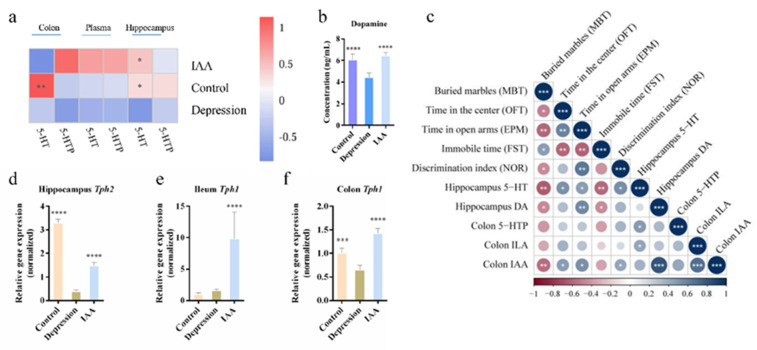
Variation of the serotonergic system and dopamine levels in mice. (**a**) Analysis of serotonin and 5-hydroxytryptophan in the plasma, colon content and hippocampus. (**b**) Concentrations of dopamine in the hippocampus of three groups. (**c**) Correlation analysis of behavioural performance and metabolites from host and gut microbiota. (**d**–**f**) Expression level of tryptophan hydroxylase-coding gene in different tissues. Data represent means and SD, compared with the depression group, * *p* < 0.05, ** *p* < 0.01, *** *p* < 0.001, **** *p* < 0.0001 compared with UCMS-treated group, one-way ANOVA followed by Tukey’s post hoc test.

An imbalance in neurotransmitter concentrations is often reported in mood disorders [13]. Pearson’s correlation analysis demonstrated that changes in 5-hydroxytryptamine (5-HT) and dopamine (DA) were positively correlated with the alleviation of anxiety and depression-like behaviours (Figure 3c). The concentrations of serotonin and DA in the hippocampus were clearly reduced in UCMS-exposed mice; this was reversed by IAA treatment (Figure 3a,b). Serotonin in the brain is metabolised by 5-HTP, which is produced through a reaction catalysed by TPH2 and the brain–gut axis. IAA administration increased the mRNA transcriptional levels of *Tph2* and upregulated the levels of *Gch1* which is an important cofactor of tryptophan hydroxylase (Figure 2h and Figure 3d). IAA observably increased the transcription of the gene encoding TPH1 in the ileum and colon by 10-fold and 3-fold, respectively, compared with UCMS-exposed mice (Figure 3e,f). Accordingly, the 5-HTP concentrations in the plasma and colon increased, whereas serotonin secretion from the colon decreased, which was different from what observed in control mice. High concentrations of enterogenous 5-HTP and enzymatic reactions in the brain may have contributed to the accumulation of brain 5-HT in the IAA group.

### 3.4. The Gut Microbiota and SCFA Concentrations Are Influenced by IAA

**Figure 4:**
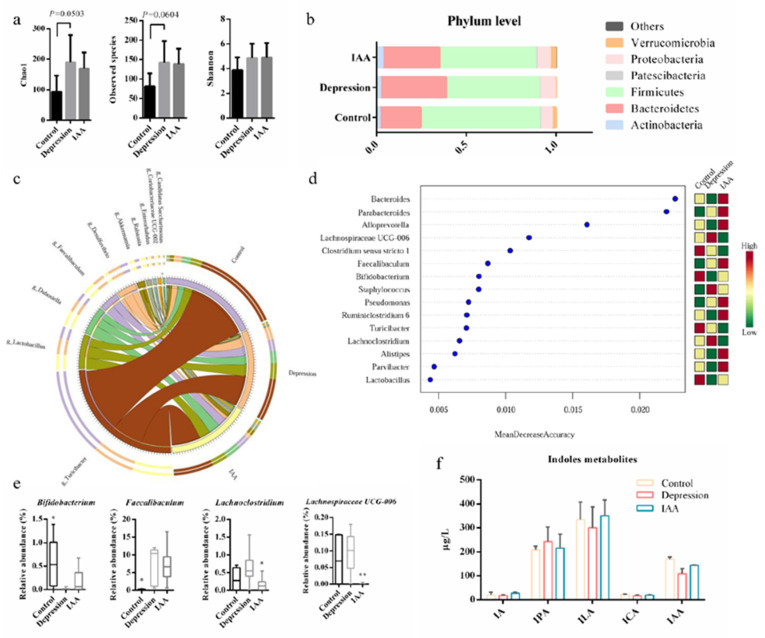
Analysis of the gut microbiota in the content of the small intestine. (**a**) Alpha diversity indicated by Chao1, Observed species and Shannon index. (**b**,**c**) Relative abundance of main bacterial communities at the phylum level and the genus level (main genera > 1%). (**d**) Differential analysis of bacterial genera in the three groups. (**e**) Relative abundance of selected differential genera. (**f**) Concentrations of indole derivatives metabolized from tryptophan in the serum. Compared with the depression group, * *p* < 0.05, ** *p* < 0.01, data are means and SD, one-way ANOVA followed by Tukey’s post-test.

Depression is almost always accompanied by an imbalance of the intestinal flora. The synthesis of 5-HT in enterochromaffin cells (EECs) has been shown to be modulated by the gut microbiota. Therefore, we investigated the effects of IAA on the bacterial composition of the small intestine and colon. Compared with the controls, the species richness of microorganisms in the small intestine of UCMS-treated mice increased; this was not alleviated by IAA gavage (Figure 4a). There was no difference in the relative proportions of Bacteroidetes, Actinobacteria and Firmicutes between UCMS and IAA groups, and the variation was contrary to that of controls (Figure 4b). Only Verrucomicrobia were similarly abundant in IAA-treated and control mice; however, the genera within Verrucomicrobia in these two groups were not clearly distinct from those in depressed mice. Genus analysis showed that *Turicibacter* was most abundant in the small intestine, followed by *Lactobacillus* (Figure 4c). Fifteen genera of the SI microbiota were found to exhibit differences between three groups. Among them, the abundances of five genera in IAA-treated mice, namely, *Parabacteroides*, *Clostridium sensu stricto* 1, *Faecalibaculum*, *Pseudomonas* and *Turicibacter*, were comparable with those in stressed mice and distinguished from those in control mice (Figure 4d). The abundances of the remaining genera were similar to those in healthy mice. Treatment of UCMS-exposed mice with IAA elicited a marked decrease in the proportions of *Lachnoclostridium* and *Lachnospiraceae* UCG-006 and a marginal increase in the change in *Bifidobacterium* abundance induced by stress (Figure 4e).

**Figure 5:**
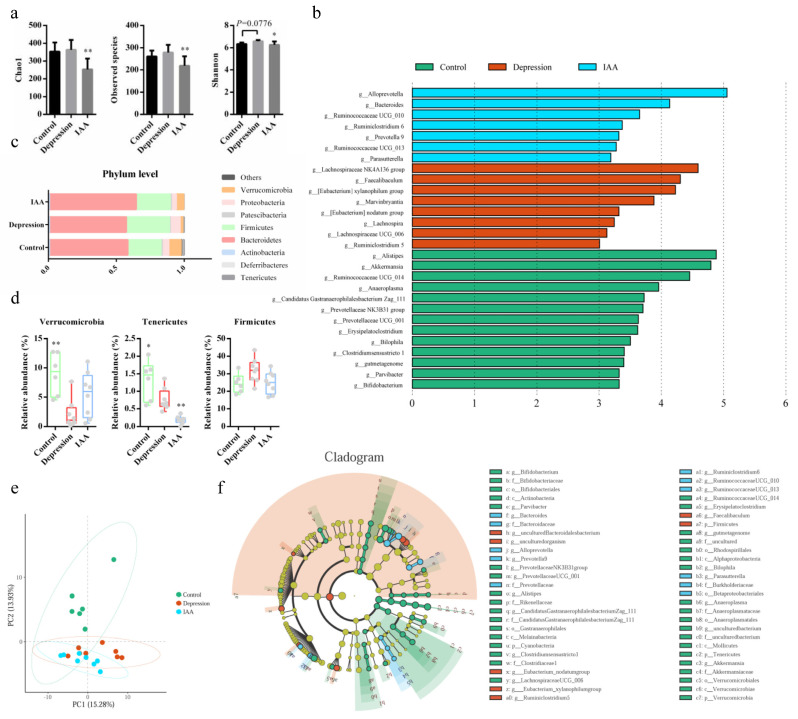
Microbial diversity in the colonic content. (**a**) Alpha-diversity analysis of the gut microbiota in the experimental groups. (**b**) LDA of bacterial composition at the genus level. (**c**,**d**) Relative abundance and STAMP analysis of microbial communities at the phylum level. Compared with the depression group, * *p* < 0.05, ** *p* < 0.01; data are presented as means and SD, one-way ANOVA followed by Tukey’s post hoc test. (**e**) PCA plot of OTU abundance. (**f**) LEFSe comparison of microorganisms among control, depression and IAA groups.

**Figure 6:**
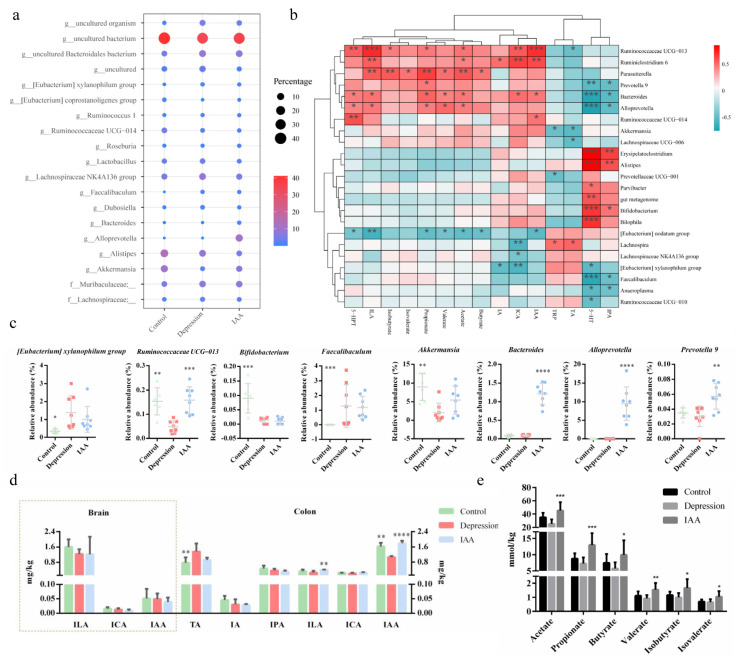
Analysis of bacterial genera in the colon content and metabolites of the gut microbiota. (**a**) Colonic microbial analysis at the genus level. (**b**) Correlation of metabolites and bacterial genera. (**c**) Differential analysis of bacteria at the genus level. (**d**) Quantification of indole derivatives in the content of colon and brain. (**e**) Concentrations of SCFA secreted from the gut microbiota in the cecum. * *p* < 0.05, ** *p* < 0.01, *** *p* < 0.001, **** *p* < 0.0001 compared with the depression group, data are presented as means ± SD, one-way ANOVA followed by Tukey’s post-test.

Similar to the alpha-diversity analysis of the gut microbiota in the small intestine, we found that UCMS induced a minor increase in the richness of microbial communities in the colon (Figure 5a). In contrast, the administration of IAA reduced the species richness. A clear separation between the control and the model groups was detected using principal component analysis (Figure 5e), indicating differences in bacterial community diversity. IAA treatment could not restore the microbial diversity to the level observed in the control group. Microbial analysis at the phylum level revealed that the relative abundances of Verrucomicrobia and Tenericutes were significantly reduced in the depression group (Figure 5c,d,f); this was reversed by IAA treatment. The control and intervention groups had lower abundances of Firmicutes and Proteobacteria than the UCMS-exposed group. Further genus analysis revealed that the colon bacteria were distinct from the small intestine bacteria (Figure 6a). UCMS increased the relative abundance of 8 genera and decreased that of 13 genera (Figure 5b). Most genera showed little variation after IAA treatment, except for *Ruminococcaceae* UCG-013 (Figure 6c). IAA treatment also significantly elevated the abundance of *Bacteroides*, *Alloprevotella* and *Prevotella* 9, which was similar between the control and the depression groups and positively correlated with the production of 5-HTP. Taken together, we found that IAA administration exerted stronger effects on the gut microbiota of the colon than on that of the small intestine. IAA treatment appeared to establish a new microbial homeostasis instead of restoring it to baseline.

SCFAs are produced by the fermentation of carbohydrates by the intestinal microbiota. A correlation analysis demonstrated that the proportions of *Ruminococcaceae* UCG-013, *Ruminiclostridium* 6, *Parasutterella*, *Prevotella* 9, *Bacteroides* and *Alloprevotella* were positively correlated with SCFA production (Figure 6b). UCMS decreased the concentrations of SCFAs compared with the control group. Nevertheless, the concentrations of six SCFAs distinctly increased upon IAA treatment.

### 3.5. IAA Affects the Tryptophan–Indoles Metabolism Pathways in the Circulation and the Brain

Essential tryptophan can be microbially metabolised into a series of indole derivatives that help maintain the intestinal homeostasis. An imbalance in the concentrations of indoles extracted from the colon content was observed in UCMS-treated mice compared with controls (Figure 6d). The depressed mice had lower concentrations of indole-3-lactic acid (ILA), indole-3-carboxaldehyde (ICA), indole-3-propionic acid (IPA) and IAA and significantly higher concentrations of tryptamine than the control mice. Exogenous supplementation with IAA remarkably elevated the concentrations of ILA and IAA, suggesting that the IAA intervention affected the tryptophan–indoles metabolism by the gut bacteria. The alleviation of abnormal emotional behaviours was positively correlated with the colon IAA and ILA concentrations (Figure 3c). However, the levels of indole derivatives in the serum were not affected by IAA administration and bacterial metabolism (Figure 4f). Fat-soluble indole derivatives can enter the brain by crossing the blood–brain barrier. The levels of detectable ILA, IAA and ICA in the hippocampus did not differ significantly across the three groups, indicating that the antidepressant effect of IAA may be independent of brain indoles and that IAA may stimulate the gut to transmit neural signals to the brain.

## 4. Discussion

Previous investigations of metabolomic signatures associated with microbes in animals and humans with psychiatric disorders highlighted the significance of the microbial metabolism of aromatic amino acids [26,27]. IAA is a tryptophan microbial metabolite that is known to be altered in the urine, cerebrospinal fluid and faeces of patients with depression [13,20,22]. However, its role in mediating depression remains unclear. This study demonstrated that the administration of IAA significantly improved anxiety and depression-like behaviours in a UCMS-induced mouse model. IAA treatment also alleviated the overactivation of the HPA axis and increased hippocampal BDNF levels. The disturbances of 5-HT and DA concentrations in the hippocampus by UCMS were alleviated by IAA supplementation. In addition, IAA reshaped the gut microbial composition in the colon, which was largely correlated with tryptophan–indoles and SCFA metabolism.

Depression is often considered a stress-related disease, as severe stressors can induce the development and increase the morbidity of depression in susceptible individuals. The physiological responses to stress are regulated by the HPA axis [2]. The normalisation of HPA axis regulation has been shown to be important for alleviating the depressive psychopathology following antidepressant treatment [28,29]. We delivered UCMS using various stressors and accordingly observed overactivation of the HPA axis, which was ameliorated by IAA. This indicated an association between alleviation of the HPA axis dysfunction by IAA and its antidepressant effects. A negative feedback to the stress response pathways is delivered by the binding of corticosterone to glucocorticoid receptors; this is compromised in the context of depression. IAA treatment elevated glucocorticoid receptor mRNA expression in the hippocampus, suggesting that IAA inhibits the release of ACTH and CRF by activating these receptors. However, the concentration of IAA in the brain was stable and unaffected by IAA treatment and UCMS exposure. This indicates that the neurobiological effects of microbial indoles may be independent of their entering the brain. In addition, IAA did not affect c-Fos expression. Thus, the molecular mechanism underlying the regulation of the HPA axis needs further exploration.

BDNF is a key molecule required for the survival and function of neurons. Stress and depression could prompt impairments of cellular resilience by reducing the BDNF levels [30,31]. An antidepressant therapy can reverse the reduction of BDNF, as indicated by the results of this study. Multiple studies have confirmed that BDNF contributes to the expression of TPH2 in the 5-HT metabolic pathway and of 5-HT receptors (5-HT1A, 5-HT2A), even the processing of serotonin-related neurons [32]. Low concentrations of serotonin have been frequently recorded in patients with depression. Most drug therapies for patients with MDD increase the intrasynaptic levels of serotonin to relieve the depressive symptoms [33]. The alleviation of depression-like behaviours by IAA may be mediated by elevated concentrations of BDNF and 5-HT in the brain. Cerebral serotonin is obtained from 5-HTP produced by tryptophan metabolism in the brain and gut. The peripheral 5-HTP and 5-HT concentrations could be increased by dietary IAA administration, as they are largely secreted by EECs in the intestinal epithelium. A previous study demonstrated that microbially derived tryptophan catabolites can stimulate EECs to promote 5-HT secretion and TPH1 expression by activating Trpa1 and deliver bacterial signals to the vagal and enteric nerves [34]. Many bacterial tryptophan catabolites, such as IAA, indole, ILA, indole acrylic acid (IA) and ICA, are ligands for the aromatic hydrocarbon receptor (AhR), which is a transcriptional regulation factor, a vital mediator of neurogenesis and astrocyte function in the brain and a regulator of immune homeostasis [35]. ILA is known to be associated with neurodevelopment in early life [36]. A recent study showed that bacterial indole could regulate adult hippocampal neurogenesis (AHN) by specifically activating AhR [18]. An imbalance in neurogenesis is an attendant phenomenon of chronic stress and depression. Regulating neurogenesis may attenuate anhedonia and HPA axis dysregulation in UCMS-exposed mice [17]. A gut microbe–indoles–AhR–neurogenesis-mediated signalling pathway could thus underlie the alleviation of depression. Our results showed that IAA treatment could alter the concentrations of various indoles, which may directly activate EECs to enhance serotonin secretion and regulate AHN by AhR activation. It has also been shown that SCFAs can increase *TPH1* mRNA levels and serotonin secretion in RIN14B cells [37]. IAA may thus affect the serotonin pathway in the gut by enhancing the production of SCFAs.

The changes in indole metabolites and SCFAs are affected by enzymatic synthesis and the composition of the intestinal microbiota. The modulation of depression and anxiety-like behaviours by IAA treatment may be associated with indole- and SCFA-producing bacteria. We found that UCMS disrupted the bacterial homeostasis and the composition of the intestinal microbiome. Increased Firmicutes and decreased Bacteroidetes in the colon, which are considered characteristics of depression susceptibility, have also been reported in several previous studies [38]. It has been reported that a lower abundance of Verrucomicrobia and Tenericute would appear in depressive mice, which is similar to our results. However, the phyla of the gut microbiota varied in previous studies. At the genus level, the abnormal relative abundance of *Lachnospiraceae*, *Turicibacter*, *Lactobacillus*, *Bifidobacterium*, *Streptococcus*, *Alistipes*, *Bacteroides*, *Prevotella*, and *Akkermansia* in individuals with depression or treated for chronic stress reported in a previous study also appeared in this study [8]. However, elevation of the [Eubacterium] xylanophilum group and *Faecalibacterium*, reported to be butyrate-producing bacteria, in UCMS-exposed mice contradicted the findings of other studies [6,39]. We did not observe an increase in butyrate levels, suggesting little to no positive effects on depression by the two genera. The composition and diversity of intestinal bacteria altered by IAA treatment were distinct from those in the UCMS-exposed group, but the alleviating effect of IAA on depression-like behaviours was not achieved by restoring the gut microbiota to previous levels. IAA treatment appeared to construct a new microbial environment and increase the proportion of bacteria producing SCFAs and indole derivatives. The *Ruminococcaceae* UCG-013, *Ruminiclostridium* 6, *Prevotella* and *Alloprevotella* strains can metabolise carbohydrates to SCFAs [40], and *Bacteroides* strains can produce tryptophan metabolites [35]. All of these genera were also correlated with the secretion of 5-HTP. Indeed, the concentrations of SCFAs and of several indoles remarkably increased with IAA administration.

## 5. Conclusions

This study illustrated that IAA supplementation alleviated depression and anxiety-like behaviours, suppressed HPA axis hyperfunction and improved the serotonergic system in a mouse model of UCMS. These effects of IAA might be associated with the modulation of the gut microbiota and microbial metabolites, such as SCFAs and tryptophan–indoles pathway components. Although the molecular mechanism by which IAA regulates the central nervous system and emotional disorders requires further exploration, this study provides a basic understanding of the antidepressant effects of IAA through the gut–brain axis and indicates the potential of microbial indole derivatives.

## Data Availability

Not applicable.

## Supplementary Materials

The following supporting information can be downloaded at: https://www.mdpi.com/article/10.3390/nu14235019/s1, Figure S1: Behavioral tests of mice injected with IAA before UCMS treatment; Table S1: The stressors applied during a typical week of the UCMS procedure; Table S2: Primer sequences for real-time PCR.

## References

[1] Joodaki M., Radahmadi M., Alaei H. (2021). Comparing the Therapeutic Effects of Crocin, Escitalopram and Co-Administration of Escitalopram and Crocin on Learning and Memory in Rats with Stress-Induced Depression. Malays. J. Med Sci..

[2] Cruz-Pereira J.S., Rea K., Nolan Y.M., O’Leary O.F., Dinan T.G., Cryan J.F. (2020). Depression’s unholy trinity: Dysregulated stress, immunity, and the microbiome. Annu. Rev. Psychol..

[3] Manji H.K., Drevets W.C., Charney D.S. (2001). The cellular neurobiology of depression. Nat. Med..

[4] Frolkis A.D., A Vallerand I., Shaheen A.-A., Lowerison M.W., Swain M.G., Barnabe C., Patten S.B., Kaplan G.G. (2019). Depression increases the risk of inflammatory bowel disease, which may be mitigated by the use of antidepressants in the treatment of depression. Gut.

[5] Chevalier G., Siopi E., Guenin-Macé L., Pascal M., Laval T., Rifflet A., Boneca I.G., Demangel C., Colsch B., Pruvost A. (2020). Effect of gut microbiota on depressive-like behaviors in mice is mediated by the endocannabinoid system. Nat. Commun..

[6] Valles-Colomer M., Falony G., Darzi Y., Tigchelaar E.F., Wang J., Tito R.Y., Schiweck C., Kurilshikov A., Joossens M., Wijmenga C. (2019). The neuroactive potential of the human gut microbiota in quality of life and depression. Nat. Microbiol..

[7] Agus A., Planchais J., Sokol H. (2018). Gut microbiota regulation of tryptophan metabolism in health and disease. Cell Host Microbe.

[8] Li S., Hua D., Wang Q., Yang L., Wang X., Luo A., Yang C. (2020). The role of bacteria and its derived metabolites in chronic pain and depression: Recent findings and research progress. Int. J. Neuropsychopharmacol..

[9] Settanni C.R., Ianiro G., Bibbò S., Cammarota G., Gasbarrini A. (2021). Gut microbiota alteration and modulation in psychiatric disorders: Current evidence on fecal microbiota transplantation. Prog. Neuro-Psychopharmacol. Biol. Psychiatry.

[10] Paiva I.H.R., Duarte-Silva E., Peixoto C.A. (2020). The role of prebiotics in cognition, anxiety, and depression. Eur. Neuropsychopharmacol..

[11] Tian P., Zou R., Wang L., Chen Y., Qian X., Zhao J., Zhang H., Qian L., Wang Q., Wang G. (2022). Multi-Probiotics ameliorate Major depressive disorder and accompanying gastrointestinal syndromes via serotonergic system regulation. J. Adv. Res..

[12] Li C.-C., Jiang N., Gan L., Zhao M.-J., Chang Q., Liu X.-M., Pan R.-L. (2020). Peripheral and cerebral abnormalities of the tryptophan metabolism in the depression-like rats induced by chronic unpredicted mild stress. Neurochem. Int..

[13] Tian P., Chen Y., Zhu H., Wang L., Qian X., Zou R., Zhao J., Zhang H., Qian L., Wang Q. (2022). Bifidobacterium breve CCFM1025 attenuates major depression disorder via regulating gut microbiome and tryptophan metabolism: A randomized clinical trial. Brain Behav. Immun..

[14] Wang D., Wu J., Zhu P., Xie H., Lu L., Bai W., Pan W., Shi R., Ye J., Xia B. (2022). Tryptophan-rich diet ameliorates chronic unpredictable mild stress induced depression-and anxiety-like behavior in mice: The potential involvement of gut-brain axis. Food Res. Int..

[15] Jaglin M., Rhimi M., Philippe C., Pons N., Bruneau A., Goustard B., Daugé V., Maguin E., Naudon L., Rabot S. (2018). Indole, a signaling molecule produced by the gut microbiota, negatively impacts emotional behaviors in rats. Front. Neurosci..

[16] Mir H.-D., Milman A., Monnoye M., Douard V., Philippe C., Aubert A., Castanon N., Vancassel S., Guérineau N.C., Naudon L. (2020). The gut microbiota metabolite indole increases emotional responses and adrenal medulla activity in chronically stressed male mice. Psychoneuroendocrinology.

[17] Eliwa H., Brizard B., Le Guisquet A.-M., Hen R., Belzung C., Surget A. (2021). Adult neurogenesis augmentation attenuates anhedonia and HPA axis dysregulation in a mouse model of chronic stress and depression. Psychoneuroendocrinology.

[18] Wei G.Z., Martin K.A., Xing P.Y., Agrawal R., Whiley L., Wood T.K., Hejndorf S., Ng Y.Z., Low J.Z.Y., Rossant J. (2021). Tryptophan-metabolizing gut microbes regulate adult neurogenesis via the aryl hydrocarbon receptor. Proc. Natl. Acad. Sci. USA.

[19] Serger E., Luengo-Gutierrez L., Chadwick J.S., Kong G., Zhou L., Crawford G., Danzi M.C., Myridakis A., Brandis A., Bello A.T. (2022). The gut metabolite indole-3 propionate promotes nerve regeneration and repair. Nature.

[20] Anderson G.M., Gerner R.H., Cohen D.J., Fairbanks L. (1984). Central tryptamine turnover in depression, schizophrenia, and anorexia: Measurement of indoleacetic acid in cerebrospinal fluid. Biol. Psychiatry.

[21] Pu J., Liu Y., Gui S., Tian L., Yu Y., Wang D., Zhong X., Chen W., Chen X., Chen Y. (2022). Effects of pharmacological treatment on metabolomic alterations in animal models of depression. Transl. Psychiatry.

[22] Zheng S., Yu M., Lu X., Huo T., Ge L., Yang J., Wu C., Li F. (2010). Urinary metabonomic study on biochemical changes in chronic unpredictable mild stress model of depression. Clin. Chim. Acta.

[23] Tian P., O’Riordan K.J., Lee Y.-K., Wang G., Zhao J., Zhang H., Cryan J.F., Chen W. (2020). Towards a psychobiotic therapy for depression: Bifidobacterium breve CCFM1025 reverses chronic stress-induced depressive symptoms and gut microbial abnormalities in mice. Neurobiol. Stress.

[24] Lefèvre A., Mavel S., Nadal-Desbarats L., Galineau L., Attucci S., Dufour D., Sokol H., Emond P. (2019). Validation of a global quantitative analysis methodology of tryptophan metabolites in mice using LC-MS. Talanta.

[25] Zhu G., Ma F., Wang G., Wang Y., Zhao J., Zhang H., Chen W. (2018). Bifidobacteria attenuate the development of metabolic disorders, with inter- and intra-species differences. Food Funct..

[26] Badawy A. (2013). Novel nutritional treatment for manic and psychotic disorders: A review of tryptophan and tyrosine depletion studies and the potential of protein-based formulations using glycomacropeptide. Psychopharmacology.

[27] Fukuwatari T. (2020). Possibility of amino acid treatment to prevent the psychiatric disorders via modulation of the production of tryptophan metabolite kynurenic acid. Nutrients.

[28] Mikulska J., Juszczyk G., Gawrońska-Grzywacz M., Herbet M. (2021). HPA Axis in the pathomechanism of depression and schizophrenia: New therapeutic strategies based on its participation. Brain Sci..

[29] Thomson F., Craighead M. (2008). Innovative approaches for the treatment of depression: Targeting the HPA axis. Neurochem. Res..

[30] Castrén E., Monteggia L.M. (2021). Brain-derived neurotrophic factor signaling in depression and antidepressant action. Biol. Psychiatry.

[31] Hing B., Sathyaputri L., Potash J.B. (2018). A comprehensive review of genetic and epigenetic mechanisms that regulate BDNF expression and function with relevance to major depressive disorder. Am. J. Med. Genet. Part B Neuropsychiatr. Genet..

[32] Popova N.K., Ilchibaeva T.V., Naumenko V.S. (2017). Neurotrophic factors (BDNF and GDNF) and the serotonergic system of the brain. Biochemistry.

[33] Weilburg J.B. (2004). An overview of SSRI and SNRI therapies for depression. Manag. Care.

[34] Ye L., Bae M., Cassilly C.D., Jabba S.V., Thorpe D.W., Martin A.M., Lu H.-Y., Wang J., Thompson J.D., Lickwar C.R. (2021). Enteroendocrine cells sense bacterial tryptophan catabolites to activate enteric and vagal neuronal pathways. Cell Host Microbe.

[35] Roager H.M., Licht T.R. (2018). Microbial tryptophan catabolites in health and disease. Nat. Commun..

[36] Saturio S., Nogacka A.M., Alvarado-Jasso G.M., Salazar N., Reyes-Gavilán C.G.D.L., Gueimonde M., Arboleya S. (2021). Role of bifidobacteria on infant health. Microorganisms.

[37] Tian P., Wang G., Zhao J., Zhang H., Chen W. (2019). Bifidobacterium with the role of 5-hydroxytryptophan synthesis regulation alleviates the symptom of depression and related microbiota dysbiosis. J. Nutr. Biochem..

[38] Qiao Y., Zhao J., Li C., Zhang M., Wei L., Zhang X., Kurskaya O., Bi H., Gao T. (2020). Effect of combined chronic predictable and unpredictable stress on depression-like symptoms in mice. Ann. Transl. Med..

[39] Xu J., Tang M., Wu X., Kong X., Liu Y., Xu X.-X. (2022). Lactobacillus rhamnosus zz-1 exerts preventive effects on chronic unpredictable mild stress-induced depression in mice via regulating the intestinal microenvironment. Food Funct..

[40] Lu S., Mikkelsen D., Yao H., Williams B.A., Flanagan B.M., Gidley M.J. (2021). Wheat cell walls and constituent polysaccharides induce similar microbiota profiles upon in vitro fermentation despite different short chain fatty acid end-product levels. Food Funct..

