# Supplementary material for: Indole Acetic Acid Exerts Anti-Depressive Effects on an Animal Model of Chronic Mild Stress
Source: Nutrients. 2022 Nov 25;14(23):5019. doi: 10.3390/nu14235019 (PMC9737131; doi:10.3390/nu14235019)
Supplement: Supplementary file 1 [file nutrients-14-05019-s001.zip › nutrients-1936431-supplementary.pdf]

Methods and Materials

Protocols of unpredictable chronic mild stress (UCMS)

The stressors of CUMS procedure were applied in a semi-random manner. According to the random number table, 1-2 stressors were randomly used every day in a week. The order of the stressors varied each week to prevent the mice from getting accustomed to it. UCMS sustained for 5 weeks. Application of the stress starts at a different time every day to minimize its predictability.

1. Water deprivation

Remove the bottle of water from the cage during 24 h. Place the bottle of water back after the time point is reached.

2. Food deprivation

Remove the food from the cage for 24 h. Place the food back.

3. Removal of bedding

Place mice in a new cage without bedding for 24 h. Return them to the cage with their mates.

4. Wet bedding

Place mice in a cage with 200 mL water per 100 g bedding for 24 h. Return them to the cage with their mates.

5. Crowding

Place mice in a small cage for 24 h. Then return them to normal cage.

6. Continuous light

Keep mice under continuous light for 24 h to disturb circadian rhythms.

7. Social isolation

Place the mouse alone in a new cage for 24 h. Return them to the cage with their mates.

8. Restraint in a drilled tube

Place the mouse in a 50 mL plastic tube, adjust it with plastic tape on the outside so the mouse is unable to move. The tube must have a hole at the far end to allow regular breathing. Hold for 3 h. Return mouse to its cage. The best way to extract the mouse is to make a sudden movement downwards, dropping it into the cage. Try to avoid pulling from the tail.

9. Forced swimming

Fill a glass tank 22 cm deep with water at 23 ± 2 °C. Place mice in the glass tank for 10 min. Then dry the mouse and return it to a clean and dry cage with fresh bedding in order to avoid chills and colds.

10. Tail clipping

The plastic clamp is clipped at 2 cm from the end of tail, sustain 3 min each time, total 3 times a day.

Behavioral tests

1. Marble burying

Mice were individually placed in a new cage filling with 5 cm thick padding. 20 marbles were put on top of the bedding in advance (5×4, marbles spaced 2 cm away from the walls and each other). After thirty minutes, the number of marbles buried for more than 2/3 of their surface was counted.

2. Novel object recognition test

Mice were placed in a white plastic square box (40×40×40 cm) with two identical objects (symmetric with respect to the mid-point of the floor) for 10 minutes. 24 hours later, a novel object substituted one of the two objects, and mice were placed in the middle of the box and monitored for 10 minutes. Directed contacts with the objects, including any contact with mouth, nose or paw, were scored. Discrimination index= (novel object exploration time-familiar object exploration time) / total exploration time.

3. Open filed

Mice were placed in the center of open arena (40×40×40 cm) and allowed to explore for 10 minutes. The time and visits of entering a virtual central zone were scored.

4. Elevated plus maze

A grey cross-shaped maze was set 1 m elevated from the floor, comprising two open and closed arms (50×5 cm, L×W). Mice were individually placed into the center of the maze, facing an open arm and allowed 5-min free exploration. The time spent and the number of entries in each arm were measured.

5. Three-chambered social approach task

The social testing apparatus was a retainage, three-chamber box. Each chamber (20×20×20 cm, L×W×H) was allowed to explore through small square openings in the dividing walls (5 cm). Two identical wire cup-like cages were placed inside each side chamber in bilaterally symmetric positions. The test has three phases: habitation of 5 min, mouse versus object, novel mouse versus familiar mouse. For the first phase, the test mouse was placed into the middle chamber, allowed to explore the whole box and empty cages inside. 5 minutes later, the test mouse was taken away from the box, and placed into the middle chamber again when object and an unfamiliar mouse were separately placed into each side cages. During phage three, the object was replaced with an unfamiliar mouse serving as a novel mouse, and the other side mouse changes into a familiar role. The time spent on contacting object, novel mouse and familiar mouse was measured.

6. Forced swim test

Mice were placed in a clear glass cylinder (30×10 cm diameter) filling with 15-cm-depth water (25±0.5℃), respectively. Water was changed between each event to remove odors. The activity of mice was monitored for 6 min. The time of immobility was detected.

7. Fear conditioning

The fear conditioning test has three phases. During phase one (conditional training), mice were individually placed into testing box and had a 3-min baseline recording, followed by 6 light/tone conditional stimulus and shock pairings with an interval of 1 min. Pairings consisted of the cue (a combined light, 260 lux) and the tone (3kHZ, 65 db) for 30 s and an electric foot shock (0.4 mA) during the last 2 s of the cue. Mice were removed from the box 2 min after the last pairing. After 24 hours, mice were placed into the box without any stimulus for 3 min, the freezing time was measured. 2 hours later, the floor and the walls of the testing box were changed into other colors, the freezing of mice was recorded under the condition of tone stimulus for 3 min and no stimulus for 1 min.

Table S1 The stressors applied during a typical week of the UCMS procedure

	Monday	Tuesday	Wednesday	Thursday	Friday	Saturday	Sunday
**08:00 a.m.**							tail clipping
**09:00 a.m.**		removal of bedding	new bedding		restraint in a drilled tube		
**10:00 a.m.**							
**12:00 a.m.**						forced swimming	tail clipping
**01:00 p.m.**				wet bedding	new bedding		
**02:00 p.m.**							
**03:00 p.m.**							tail clipping
**08:00 p.m.**<br>**Overnight**	food deprivation		water deprivation	continuous light	crowding		social isolation

Table S2 Primer sequences for real-time PCR

Gene	Forward primer (5′-3′)	Reverse primer (5′-3′)
Tph1	CACAGTTCAGATCCCCTCTACA	GAACGTGGCCTAGGAGTTCA
Tph2	CTGAATCCGCCTGAGAGCAT	CCGTACATGAGGACTCGGTG
Gch1	TGCTTACTCGTCCATTCTGC	CCTTCACAATCACCATCTCG
Nr3c1	CCGGGTCCCCAGGTAAAGA	TGTCCGGTAAAATAAGAGGCTTG
Nr3c2	GAAAGGCGCTGGAGTCAAGT	TGTTCGGAGTAGCACCGGAA
IL-6	GAGGATACCACTCCCAACAGACC	AAGTGCATCATCGTTGTTCATACA
IL-22	TGGGATTTGTGTGCAAAAGCA	TAATTTCCAGTCCTGTCTTCTG
TNF-α	ATGAGCACAGAAAGCATGATC	TACAGGCTTGTCACTCGAATT
Gapdh	AGGTCGGTGTGAACGGATTTG	TGTAGACCATGTAGTTGAGGTCA

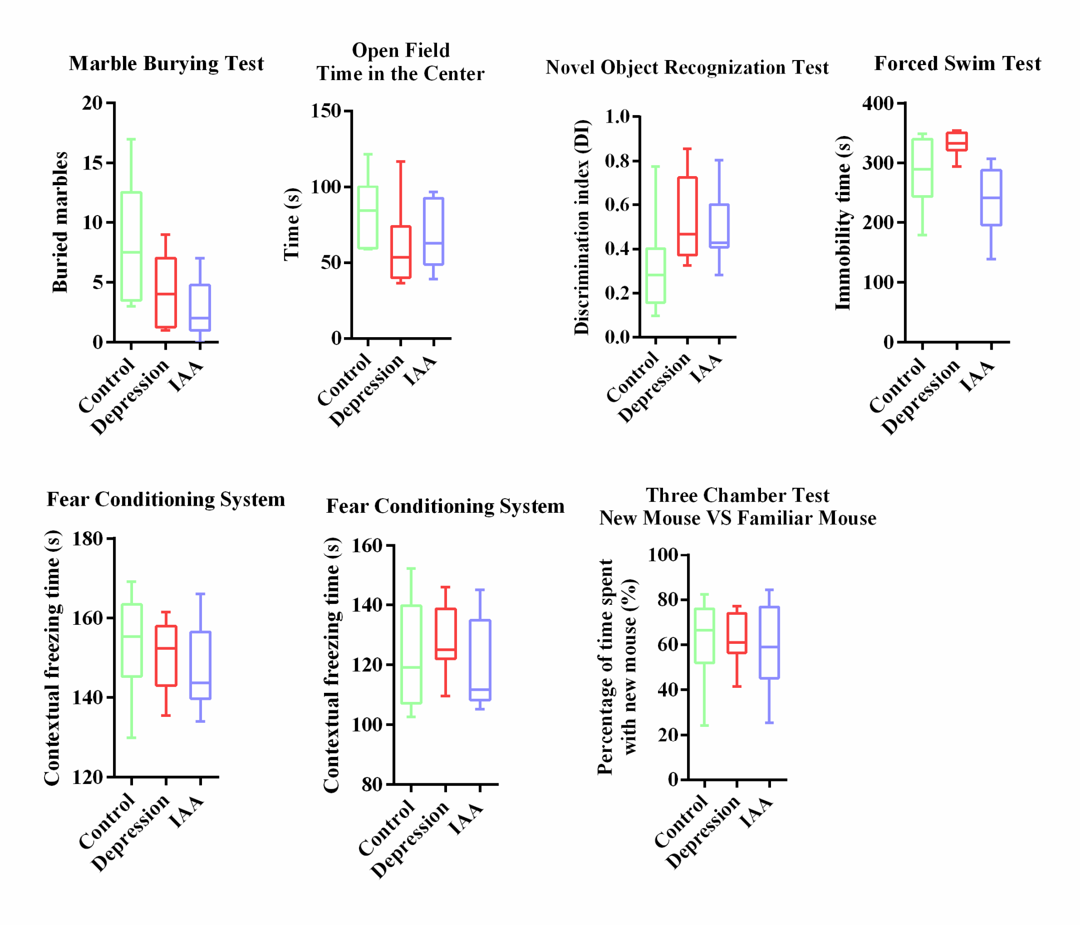

Figure S1. Behavioural tests of mice injected with IAA before UCMS treatment.
